# Multifunctional Nutraceutical Composition Based on Fermented Spirulina, Apple Cider Vinegar, Jerusalem Artichoke, and Bovine Colostrum

**DOI:** 10.3390/foods12081690

**Published:** 2023-04-18

**Authors:** Elena Bartkiene, Vytaute Starkute, Ieva Jomantaite, Egle Zokaityte, Ernestas Mockus, Ernesta Tolpeznikaite, Gintare Zokaityte, Penka Petrova, Antonello Santini, João Miguel Rocha, Fatih Özogul, Dovile Klupsaite

**Affiliations:** 1 Department of Food Safety and Quality, Veterinary Academy, Lithuanian University of Health Sciences, Tilzes Str. 18, LT-47181 Kaunas, Lithuania; elena.bartkiene@lsmuni.lt (E.B.); vytaute.starkute@lsmuni.lt (V.S.); ieva.jomantaite@stud.lsmu.lt (I.J.); 2 Institute of Animal Rearing Technologies, Faculty of Animal Sciences, Lithuanian University of Health Sciences, Tilzes Str. 18, LT-47181 Kaunas, Lithuania; egle.zokaityte@lsmuni.lt (E.Z.); ernestas.mockus@lsmuni.lt (E.M.);; 3 Institute of Microbiology, Bulgarian Academy of Sciences, Acad. G. Bontchev Str. bl. 26, 1113 Sofia, Bulgaria; 4 Department of Pharmacy, University of Napoli Federico II, Via D. Montesano 49, 80131 Napoli, Italy; asantini@unina.it; 5 CBQF-Centro de Biotecnologia e Química Fina—Laboratório Associado, Escola Superior de Biotecnologia, Universidade Católica Portuguesa, Rua Diogo Botelho 1327, 4169-005 Porto, Portugal; jmfrocha@fc.up.pt; 6 LEPABE—Laboratory for Process Engineering, Environment, Biotechnology and Energy, Faculty of Engineering, University of Porto, Rua Dr. Roberto Frias, s/n, 4200-465 Porto, Portugal; 7 ALiCE—Associate Laboratory in Chemical Engineering, Faculty of Engineering, University of Porto, Rua Dr. Roberto Frias, s/n, 4200-465 Porto, Portugal; 8 Department of Seafood Processing Technology, Faculty of Fisheries, Cukurova University, Balcali, Adana 01330, Turkey; fozogul@cu.edu.tr; 9 Biotechnology Research and Application Center, Cukurova University, Balcali, Adana 01330, Turkey

**Keywords:** nutraceuticals, Spirulina, fermentation, lactic acid bacteria, apple cider vinegar, Jerusalem artichoke, bovine colostrum

## Abstract

The main purpose of this experiment was to develop a multifunctional nutraceutical composition based on ingredients of different origins (Spirulina powder (SP), bovine colostrum (BC), Jerusalem artichoke powder (JAP), and apple cider vinegar (ACV)) which possess different health benefits through their different mechanisms of action. In order to improve the functional properties of Spirulina and bovine colostrum, fermentation with the *Pediococcus acidilactici* No. 29 and *Lacticaseibacillus paracasei* LUHS244 strains, respectively, was carried out. These LAB strains were chosen due to their good antimicrobial properties. The following parameters were analysed: for Spirulina (non-treated and fermented)—pH, colour coordinates, fatty acid profile, and contents of L-glutamic and GABA acids; for bovine colostrum (non-treated and fermented)—pH, colour coordinates, dry matter, and microbiological parameters (total LAB, total bacteria, total enterobacteria, *Escherichia coli*, and mould/yeast counts); for the produced nutraceuticals—hardness, colour coordinates, and overall acceptability. It was established that fermentation reduced the pH of the SP and BC and affected their colour coordinates. Fermented SP contained a greater concentration of gamma-aminobutyric and L-glutamic acids (by 5.2 times and 31.4% more, respectively), compared to the non-treated SP and BC. In addition, the presence of gamma-linolenic and omega-3 fatty acids was observed in fermented SP. Fermentation of BC reduces *Escherichia coli*, total bacteria, total enterobacteria, and total mould/yeast counts in samples. The obtained three-layer nutraceutical (I layer—fermented SP; II—fermented BC and JAP; III—ACV) demonstrated a high overall acceptability. Finally, our finding suggest that the selected nutraceutical combination has immense potential in the production of a multifunctional product with improved functionality and a high acceptability.

## 1. Introduction

A nutraceutical is a biologically active compound, dietary supplement, or food that delivers health advantages, including the ability to prevent and treat illnesses [1]. The public interest in natural nutraceutical items is significantly increasing because natural items are preferred by consumers, and the consumption of these products does not require a prescription from a doctor [2,3,4]. It was reported that the global market for nutraceuticals is expected to reach USD 578.23 billion by 2025 [5].

The development of a nutraceutical in the form of a gummy confection has great potential because this type of product is attractive to consumers due to its confectionary appearance and taste [6]. The chewable gummy market in the United States is expected to reach USD 5.8 billion by 2029 [7]. Formulations of gummy candies or chewable tablets with health-promoting ingredients have already been reported [6,8,9,10]. However, these ingredients induce various effects on the product’s physicochemical characteristics and acceptability; therefore, the demand for new formulations with better sensory properties is still increasing. There are many potential ingredients for the development of nutraceutical combinations, e.g., Spirulina powder, bovine colostrum, Jerusalem artichoke powder, apple cider vinegar, etc.

Spirulina is an edible cyanobacterium with significant potential for improving human health [11]. These microalgae have a GRAS (generally regarded as safe) status and are called a “superfood”. The protein content in Spirulina is high (up to 60–70%), and the percentages of saturated, polyunsaturated (1.50–2.00%), and essential fatty acids (5.00–8.20%) are also high [12]. Spirulina’s fatty acid profile includes γ-linolenic acid and α-linoleic acid. Spirulina is rich in vitamins (e.g., vitamins B12, K, and E) and minerals (Ca, K, Fe, Na, Zn, etc.) [13]. The majority of the phytonutrients and certain flavonoid compounds (isoflavones, dihydrochalcones, flavanones, and flavonols) in Spirulina have strong antioxidant capabilities [12]. The well-known pigment chlorophyll in Spirulina is beneficial due to its antioxidant and antimutagenic activities, while the main pigment phycocyanin possesses antioxidative, anti-inflammatory, anticarcinogenic, and hepatoprotective effects [11]. In general, the nutraceutical properties of Spirulina include detoxifying, nourishing, antioxidant, immuno-stimulatory, anti-obesity, anti-diabetic, anti-aging, neuroprotective, anti-inflammatory, and anti-microbial effects [12].

Bovine colostrum is the milk produced from a cow’s mammary glands immediately after calving. A distinctive combination of nutrients and bioactive substances can be found in bovine colostrum [14]. Colostrum contains immunoglobulins, α- and β-lactalbumin, growth factors, oligosaccharides and glycosylated proteins (which act as prebiotics), and vitamins A, D, E, and B-group [15]. The immunoglobulins, bioactive oligosaccharides, lactoperoxidase, lysozyme, and lactoferrin in colostrum induce antimicrobial effects, whereas it scytokines, leukocytes, and colostrinin are involved in immune function [14]. The numerous immunomodulating components that bovine colostrum contains, which uphold and restore the health of the gastrointestinal tract, are primarily responsible for its favourable effects on the prevention and treatment of gastrointestinal illnesses (inflammatory bowel disease, diarrhoea, constipation, short bowel syndrome, and colon issues) [16]. It is safe for humans to consume and there are no contraindications, and only several side effects have been reported [15]. 

Jerusalem artichoke (*Helianthus tuberosus* L.) contains the bioactive compound inulin and essential amino acids such as leucine, methionine + cystine, isoleucine, lysine, histidine valine, threonine, etc. [17]. Inulin can act as a prebiotic and has been shown to be an antimicrobial and anti-inflammatory agent and a wound healer [18]. Some proteins in *H. tuberosus* are important for the control of Alzheimer’s, Huntington’s, and Parkinson’s diseases, while others (Kunitz-type and serine hydroxy-methyltransferase proteins) possess antimicrobial and anticancer effects [17]. Fructooligosaccharides found in *H. tuberosus* have nutraceutical properties, boost immunity, and improve intestinal flora, blood lipids, and bone health [19].

Apple cider vinegar, which is used to preserve and flavour foods, contains organic acids (acetic and malic acids), flavonoids (quercetin, kaempferol, gallic, ferulic, caffeic acids, and catechin), polyphenols, minerals, and vitamins [20]. Due to its antimicrobial, antioxidant, and antibiotic actions, and its ability to maintain the acid–base balance in blood, apple cider vinegar plays an important part in regulating weight loss and preventing body and joint pains, cancer, diabetes, and cardiovascular diseases [21]. 

The above-mentioned benefits make these ingredients valuable materials for nutraceutical production. However, since nutraceuticals can be concentrated versions of food, it is likely that the level of some unwanted compounds (e.g., pesticides, mycotoxins, heavy metals, etc.) could be increased in a product after the extraction process [22]. Therefore, the bioconversion, e.g., fermentation with lactic acid bacteria (LAB), of separate ingredients could not only enhance their functional properties but also increase their safety [23]. LAB, which has a GRAS status, possess antimicrobial and enzymatic activities, improves sensory properties, prolongs the storage duration of products, and acts as a probiotics In addition to the synthesis of exopolysaccharides, organic acids, bacteriocins, etc., LAB can produce γ-amino butyric acid (GABA) [24]. The glutamate decarboxylase in LAB cells is in charge of GABA synthesis, and many LAB with this enzyme activity have been identified, including *Lactobacillus*, *Pediococcus*, and *Lactococcus* strains [25]. The precursor of GABA is L-glutamic acid, which is a flavour enhancer and neurotransmitter [26]. GABA is a key inhibitory neurotransmitter and has already been recognized as antidepressant, immunity booster, regulator of blood pressure, and an anti-hypertension, anti-diabetic, and anticarcinogenic agent [25]. However, GABA levels in fermented foods depend on the conditions of the fermentation process [27]. Our previous studies showed that fermentation with selected LAB strains is suitable technology for increasing the concentration of GABA in Spirulina [28]. Additionally, our previous studies showed that fermenting bovine colostrum improves it safety characteristics and functional properties, including its antimicrobial properties [29,30]. 

Finally, taking into consideration that (I) Spirulina shows detoxifying, antioxidant, immuno-stimulatory, anti-obesity, anti-diabetic, anti-aging, neuroprotective, anti-inflammatory, and anti-microbial effects, (II) bovine colostrum could help to restore gastrointestinal tract functions, (III) *H. tuberosus* L. shows antimicrobial and anti-inflammatory properties, boosts immunity, and improves intestinal flora, and (IV) apple cider vinegar, due to antimicrobial and antioxidant actions, plays an important role in the prevention of many diseases the combination of these ingredients has many prospects for the development of a nutraceutical formula.

Therefore, the main purpose of this experiment was to develop a multifunctional nutraceutical composition based on ingredients of different origins (Spirulina powder, bovine colostrum, Jerusalem artichoke powder, and apple cider vinegar) that possess different health benefits through their different mechanisms of action. In order to improve the functional properties of Spirulina and bovine colostrum, fermentation with *Pediococcus acidilactici* No. 29 and *Lacticaseibacillus paracasei* LUHS244 strains, respectively, was carried out. These LAB strains were chosen due to their good antimicrobial properties, which were analysed in our previous work [31]. The following parameters were analysed: for Spirulina (non-treated and fermented)—pH, colour coordinates, fatty acid profile, and contents of L-glutamic and GABA acids; for bovine colostrum (non-treated and fermented)—pH, colour coordinates, dry matter, and microbiological parameters (total LAB, total bacteria, total enterobacteria, *Escherichia coli*, and mould/yeast counts); for produced nutraceuticals—the hardness, colour coordinates, and overall acceptability.

## 2. Materials and Methods

### 2.1. Materials Used for Multifunctional Nutraceutical Preparation

Lyophilized Spirulina (*Arthrospira platensis*) powder (content per 100 g: 1.1 g of sodium, 30.3 g of total carbohydrates, 60.6 g of proteins, 151.5 mg of calcium, 1.7 mg of potassium, and 48.5 mg of iron) was provided by Now Foods Company (Bloomingdale, IL, USA).

Apple cider vinegar was obtained from the agricultural company Auseklis (Ardiskis, Lithuania).

Jerusalem artichoke powder (content per 100 g: <0.01 g of sodium, 81.7 g of total carbohydrates (comprising 27.6 g of sugar, 13.3 g of dietary fibre, and 46.1 g of inuline), 10.0 g of protein, 160 mg of calcium, 73.0 mg of magnesium, 3.26 mg of iron, and 0.3 mg of vitamin B6) was obtained from the Ltd. Urban Food (Kėdainiai, Lithuania) and producer JSC Kvalitetas (Dotnuva, Lithuania).

The bovine colostrum samples were collected from the Bentnoriaus agricultural company (Paliepiu village, Lithuania). This agricultural company maintains Lithuanian black-and-white (Holstein) dairy cows. Bovine colostrum was collected during the spring period of the year 2022. At the agricultural company, bovine colostrum was collected within 2 h of calf delivery and kept frozen at −18 °C before use.

### 2.2. Spirulina Fermentation and Analysis Methods

*Pediococcus acidilactici,* strain No. 29, was acquired from the collection of the Lithuanian University of Health Sciences (Kaunas, Lithuania). Before the experiment, *Pediococcus acidilactici*, strain No. 29, was incubated and multiplied in De Man, Rogosa, and Sharpe (MRS) broth culture medium (Biolife, Milano, Italy) at 30 °C under anaerobic conditions for 24 h. 

A total of 3 mL of fresh *Pediococcus acidilactici*, strain No. 29, grown in MRS broth (at an average cell concentration of 8.6 log_10_ CFU/mL), was inoculated in 100 mL of Spirulina/water mixture (Spirulina/water ratio of 1:2, *w*/*w*) and fermented at 30 °C under anaerobic conditions for 24 h.

In the later stages of the experiment, Spirulina parameters (pH, colour coordinates, L-glutamic and GABA concentrations, and fatty acid profile) were evaluated.

The pH of the Spirulina/water mixture was evaluated using a pH meter (Inolab 3, Hanna Instruments, Venet, Italy) by inserting the pH electrode into the samples. The colour coordinates of the Spirulina were evaluated on the sample surface using the International Commission on Illumination (CIE) L*a*b* colour space system (CromaMeter CR-400, Konica Minolta, Marunouchi, Tokyo, Japan).

Analyses of the concentrations of L-glutamic acid (L-Glu) and GABA in the Spirulina were carried out using a TSQ Quantiva MS/MS coupled to a Thermo Scientific Ultimate 3000 HPLC instrument (Thermo Scientific, Waltham, MA, USA). All methods are described by Tolpeznikaite et al. in detail [28]. An analysis of the fatty acid (FA) profile of the Spirulina samples was performed using a gas chromatograph GC 2010 Plus (Shimadzu Europa GmbH, Duisburg, Germany) equipped with a mass spectrometer, GC-MS QP2010 (Shimadzu Europa GmbH, Duisburg, Germany). All methods are described by Tolpeznikaite et al. in detail [32].

### 2.3. Bovine Colostrum Fermentation and Analysis Methods

The LUHS244 strain of *Lacticaseibacillus paracasei* was chosen according to its proper antibacterial and antifungal properties [31]. Before the experiment, *Lc. paracasei* was stored at −80 °C in a Microbank system (Pro-Lab Diagnostics, UK) and grown in de Man, Rogosa, and Sharpe (MRS) broth (CM 0359, Oxoid, Hampshire, UK) at 30 °C for 48 h prior to use. An amount of 3 mL of MRS broth containing multiplied *Lc. paracasei* (at an average LAB cell concentration of 9.2 log_10_ CFU/mL) was inoculated into 100 mL of defrosted (at 24 ± 2 °C for 12 h) bovine colostrum, followed by fermentation in a CO_2_ incubator (Memmert GmbH + Co. KG, Schwabach, Germany) for 24 h at 30 °C. 

The pH of the bovine colostrum was evaluated using a pH meter (Inolab 3, Hanna Instruments, Venet, Italy) by inserting the pH electrode into the samples. The colour coordinates of the bovine colostrum were evaluated on the sample surface using the International Commission on Illumination (CIE) L*a*b* colour space system (CromaMeter CR-400, Konica Minolta, Marunouchi, Tokyo, Japan). The dry matter (DM, %) of the bovine colostrum samples was measured with a Pal-3 refractometer (Atago, Japan).

The microbiological parameters of the bovine colostrum were also evaluated (total LAB, total bacteria, total enterobacteria, *Escherichia coli*, and mould/yeast counts). For the evaluation of the total LAB count, 10 g of bovine colostrum was homogenized with 90 mL of saline (9 g/L NaCl solution). Serial dilutions of 10^−4^–10^−8^ with saline were used for the sample preparation. Sterile MRS (Man, Rogosa, and Sharpe) agar (CM0361, Oxoid) with a thickness of 5 mm was used for bacterial growth on Petri dishes. The dishes were separately seeded with the sample suspension using surface sowing and incubated under anaerobic conditions at 30 °C for 72 h. The total bacteria count was determined using plate count agar (PCA), and the bacteria were incubated under aerobic conditions at 32 °C for 24–48 h (CM0325, Oxoid, UK). MacConkey (Oxoid Ltd., Basingstoke, United Kingdom) and Tryptone Bile X-glucuronide agar (Oxoid Ltd., Basingstoke, United Kingdom) were used for the determination of the total number of enterobacteria and *Escherichia coli* (at 35–37 °C for 18–24 h). The mould and yeast were determined on chloramphenicol agar (CM0549, Oxoid, UK) after incubation at 25 ± 2 °C for 5 days. The number of microorganisms was counted and expressed as log_10_ of colony-forming units per millilitre (CFU/mL).

### 2.4. Nutraceutical Preparation and Analysis Methods

**Figure 1:**
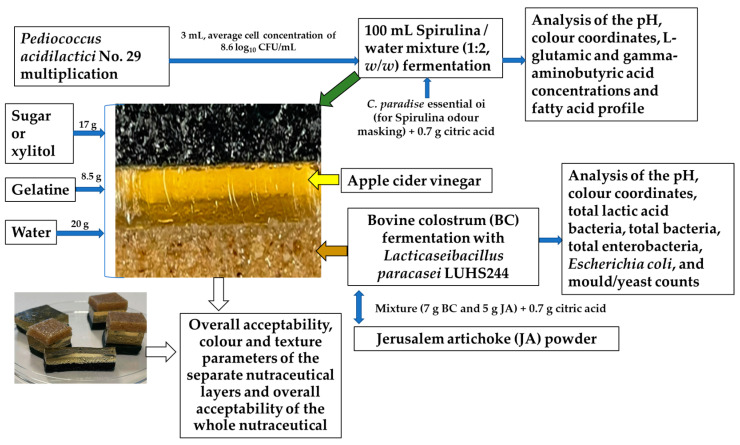
Principal scheme of the experiment.

Gelatine (Klingai, Lithuania) was used for nutraceutical texture formation. Xylitol (Natur Hurtig, Nuremberg, Germany), citric acid (Sanitex, Kaunas, Lithuania), and sugar (Nordic Sugar Kėdainiai, Kedainiai, Lithuania) were purchased at a local market (JSC Maxima LT, Kaunas, Lithuania). Grapefruit (*Citrus paradise*, producer JSC Zolotonošskaja PKF, Komunarovskaja, Ukraine) essential oil was obtained from JSC Gintarine vaistine (Kaunas, Lithuania) and was used as an odour-masking agent for the nutraceuticals with a Spirulina formulation. The formulas of the nutraceutical groups are shown in Table 1. Furthermore, in the formulation of the nutraceuticals, sugar was exchanged for xylitol. The principal scheme of the experiment is shown in Figure 1.

For the preparation of the nutraceuticals, gelatine powder was first soaked in water for 30 min and then melted by heating for 15 min at 90 °C. Sugar or xylitol was added and dissolved in the mixture while boiling. The obtained mixture was further heated to 90 °C under stirring. According to the provided recipes, other ingredients (citric acid, fermented Spirulina, essential oil, fermented bovine colostrum, Jerusalem artichoke, and apple cider vinegar) were incorporated into the nutraceutical mass at the end of the process (mass temperature 40 °C). The mass obtained after mixing was poured into a cast, and the nutraceuticals were dried at 22 ± 2 °C for 24 h to achieve a hard-gel form.

The hardness of the separate layers of the nutraceuticals was evaluated by the texture profile analysis (TPA), using a Texture Analyser TA.XT2 (StableMicro Systems Ltd., Godalming, UK) (compression force—0.5 N; test speed—0.5 mm/s; post-test speed—2 mm/s; distance—6 mm). The colour coordinates of the separate nutraceutical layers were evaluated on the sample surfaces using the International Commission on Illumination (CIE) L*a*b* colour space system (CromaMeter CR-400, Konica Minolta, Marunouchi, Tokyo, Japan). A sensory analysis of the separate nutraceutical layers and the whole product was carried out according to the ISO 6658 method [33]. Thirty panellists evaluated the overall acceptability (OA) of the nutraceuticals using the hedonic scale from 0 (dislike extremely) to 10 (like extremely).

### 2.5. Statistical Analysis

The fermentation of the Spirulina samples was performed in duplicate, and all analytical experiments were carried out in triplicate (n = 6). Bovine colostrum analyses were carried out in triplicate (n = 3). The preparation of the nutraceuticals was performed in duplicate, and analyses of the colour coordinates and texture hardness were carried out in triplicate (n = 6). The overall acceptability of the samples was evaluated by thirty panellists. The mean values were calculated using the statistical package IBM^®^ SPSS^®^ for Windows (v28.0.1.0 (142), SPSS, Chicago, IL, USA). The data were compared using Duncan’s multiple range test, with significance defined at *p* ≤ 0.05. A linear Pearson correlation was used to quantify the strength of the relationship between the variables. The results were recognized as statistically significant at *p* ≤ 0.05.

## 3. Results and Discussion

### 3.1. Parameters of Spirulina (pH, Colour Coordinates, L-Glutamic and Gamma-Aminobutyric Acids Concentrations and Fatty Acid Profile)

**Figure 2:**
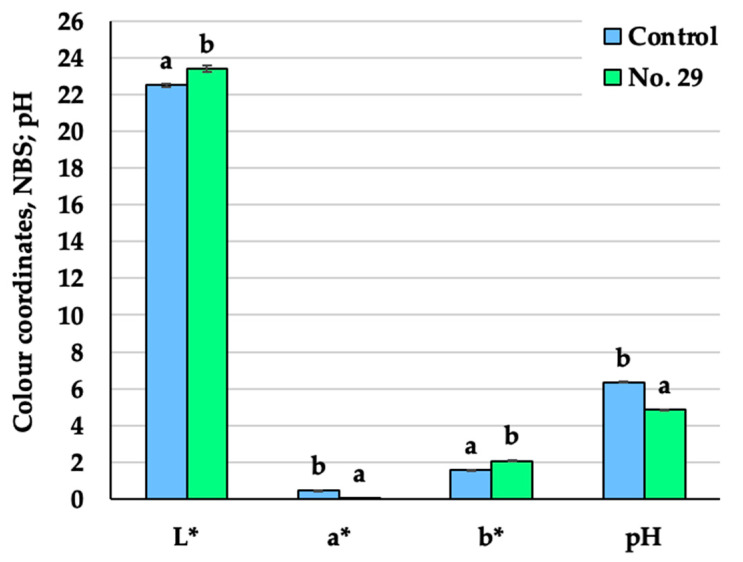
Changes in the pH values and colour coordinates in the Spirulina samples (Control—Spirulina powder and water mixture, 1:2 *w*/*w*; No. 29—Spirulina samples fermented for 24 h with *Pediococcus acidilactici,* strain No. 29; L*—lightness; a*—redness or—a*—greenness; b*—yellowness or—b*—blueness; NBS—National Bureau of Standards units. Data are represented as means (n = 6) ± standard error. a–b mean values denoted with different letters indicate significantly different values between the samples (*p* ≤ 0.05)).

The colour coordinates (L*, a* and b*) and pH values of the non-treated Spirulina and the Spirulina fermented with *P. acidilactici* are provided in Figure 2. The L* (lightness) and b* (yellowness) coordinate values were significantly higher (by 4 and 32.9% on average, respectively) in the fermented Spirulina when compared to the non-treated samples. On average, the value of the a* coordinate (redness) was 8.6 times lower in the fermented Spirulina than the non-treated samples. A decrease in pH was observed in the fermented Spirulina, and its pH value was lower by 23.5% on average when compared to the non-treated Spirulina.

Chlorophyll, which predominates in Spirulina, gives the samples their dark green colour, while carotenoids and C-phycocyanin may be related with the presence of redness and blueness [34]. The changes in the colour coordinates of the fermented Spirulina could be related to the fact that bound pigments are released as a result of the decrease in pH and enzyme activity during the fermentation process [23]. Spirulina contains fermentable sugars such as rhamnose, glucose, galactose, etc., which serve as a good substrate for the growth of LAB [35]. The bacterial synthesis of organic acids, especially lactic acid, causes a drop in pH during fermentation [23]. Similar results of the pH drop in fermented Spirulina were also found by de Marco Castro et al. and Bao et al. [23,36].

**Figure 3:**
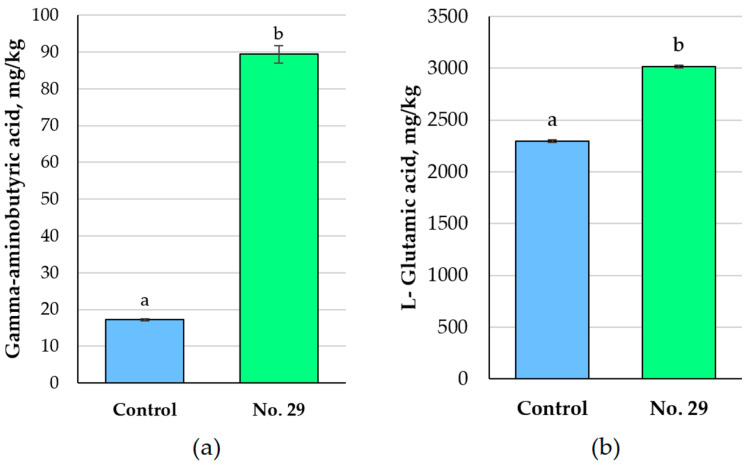
Concentrations of gamma-aminobutyric acid (GABA) (**a**) and L-Glutamic acid (L-Glu) (**b**) in the Spirulina samples (Control—Spirulina powder and water mixture, 1:2 *w*/*w*; No. 29—Spirulina samples fermented with *Pediococcus acidilactici,* strain No. 29, for 24 h. Data are represented as means (n = 6) ± standard error. a–b mean values denoted with different letters indicate significantly different values between the samples (*p* ≤ 0.05)).

The concentrations of GABA and L-Glu in the Spirulina samples (non-treated and fermented) are provided in Figure 3. The fermented Spirulina had greater concentrations of both GABA and L-Glu (by 5.2 times and 31.4%, respectively) compared to the non-treated samples.

A non-essential amino acid called L-Glu can be found in a wide range of foods in both its free and protein-bound forms [37]. It is mainly produced through microbial fermentation, and the proteolytic enzyme L-glutaminase is involved in the hydrolysation of L-glutamine to produce L-Glu [38]. Our results showed an increase in the content of L-Glu after Spirulina fermentation that is probably related to the proteolytic activity of *P. acidilactici*. GABA is thought to be a powerful bioactive agent with a wide range of physiological effects, including anti-stress, pain-relieving, antioxidant, and anti-insomnia effects [39]. Microorganisms (bacteria, yeasts, and moulds) produce this compound from L-Glu by glutamate decarboxylase, but the ability of various species to generate GABA differs considerably [25]. The glutamate decarboxylase is found in a variety of LAB species, including *Pediococcus, Lactobacillus*, and *Lactococcus*. Anggraini et al. [40] reported GABA production by *Pediococcus acidilactici* DS15 using tofu water and palm sugar as sources of nitrogen and carbon. The GABA-producing capacity of *Pedioccocus pentosaceus* MN12, isolated from fermented fish sauce, was also examined [41]. The results of our study also indicated the ability of *P. acidilactici* No. 29 to produce GABA during the fermentation of Spirulina. However, factors such as acidity, L-Glu content, duration of fermentation, temperature, and medium composition strongly influence the rates of GABA production [27].

**Figure 4:**
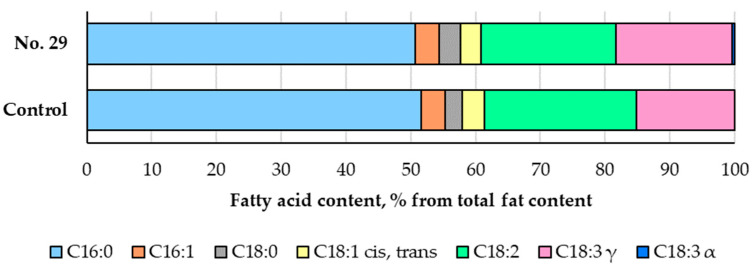
Fatty acid (FA) profile in the Spirulina samples (Control—Spirulina powder and water mixture, 1:2 *w*/*w*; No. 29—Spirulina samples fermented for 24 h with *Pediococcus acidilactici,* strain No. 29; C16:0—palmitic acid; C16:1—palmitoleic acid; C18:0—stearic acid; C18:1 *cis-* and *trans*-9-oleic acid; C18:2—linoleic acid; C18:3γ—gamma-linolenic acid; C18:3α—alfa linolenic acid. Data are represented as means (n = 6) ± standard error. a–b mean values denoted with different letters indicate significantly different values between the samples (*p* ≤ 0.05)).

**Figure 5:**
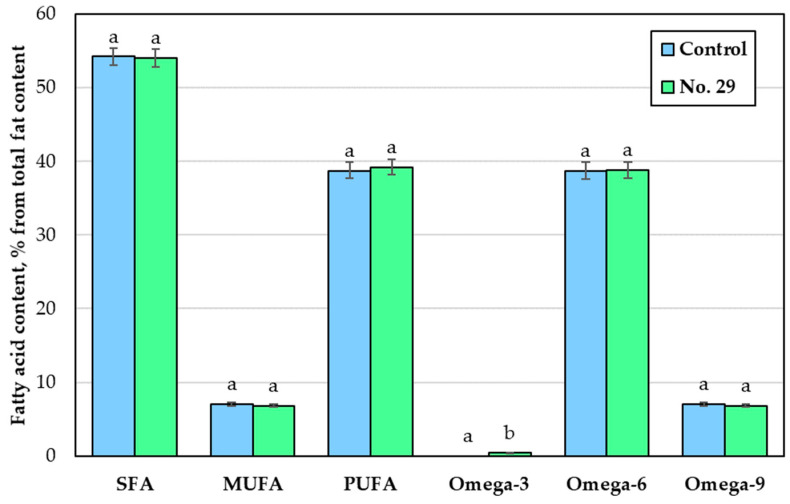
Classification of fatty acids (FA) in the Spirulina samples (Control—Spirulina powder and water mixture, 1:2 *w*/*w*; No. 29—Spirulina samples fermented for 24 h with *Pediococcus acidilactici,* strain No. 29; SFAs—saturated fatty acids; MUFAs—monounsaturated fatty acids; PUFAs—polyunsaturated fatty acids; omega 3—omega 3 fatty acids; omega 6—omega 6 fatty acids; omega 9—omega 9 fatty acids; Data are represented as means (n = 6) ± standard error. a–b mean values denoted with different letters, indicates significantly different values between the samples (*p* ≤ 0.05)).

The fatty acid (FA) profile and classification of the non-treated and fermented Spirulina samples are presented in Figure 4 and Figure 5. It was found that palmitic (C16:0) acid composed half of the total FA content in all samples. Palmitic acid, gamma linolenic (C18:3γ), and linoleic (C18:2) acids were the predominant FAs in both the non-treated and fermented Spirulina. On average, the fermented samples contained 2.4%, 3%, and 11.1% less palmitoleic (C16:1), *cis-* and *trans*-9-oleic (C18:1 *cis*, *trans*), and linoleic (C18:2) acids, respectively, than the non-treated Spirulina. However, these samples had increased levels of stearic (C18:0) and alpha-linolenic (C18:3α) acids by 22.8% and 17.8%, respectively, compared to the non-treated samples. The presence of gamma-linolenic (C18:3γ) acid was observed after fermentation with *P. acidilactici*, while the content of C16:0 was similar in both types of samples. The main components of the entire FA profile were saturated (SFA), polyunsaturated (PUFA), and omega-6 fatty acids. Non-treated and fermented Spirulina had similar levels of SFA (on average, 54%), monounsaturated (MUFA) (on average, 7%), PUFA (on average, 39%), omega-6 (on average, 39%), and omega-9 (on average, 7%) fatty acids. However, the presence of omega-3 fatty acids was observed after the fermentation of Spirulina.

Spirulina is a great source of PUFA and contains high concentrations of essential FAs, particularly γ-linolenic acid. In the literature, the data on the FA profile of Spirulina varies due to different Spirulina species, countries of origin, growing conditions, and development stages at the time of harvest [42]. One study revealed that the amounts of SFA, PUFA, MUFA, and omega-6 in *Arthrospira platensis* accounted for 62.9–80.3%, 10.6–24.5%, 9–13%, and 10–24%, respectively, of the total FAs, and these numbers were influenced by the Spirulina’s country of origin [43]. Compared to these results, we found slightly higher levels of PUFA and omega-6, lower level of SFA, and a similar level of MUFA in the non-treated Spirulina. Moreover, similar to our results, that research study did not detect omega-3 or find it in small amounts. It was reported that the percentage of palmitic acid (16:0) in *Arthrospira platensis* is 25% of the lipids [44]. However, we observed a higher content of C16:0 in the non-treated Spirulina. The changes in the FA profile after fermentation could be related to the ability of LAB to utilize lipids and synthesize secondary metabolites in a fermented substrate (e.g., FAs) [45,46]. Similar to our results, in the study by Dewi and Amalia, Spirulina was fermented with *Lactiplantibacillus plantarum* (FNCL 0127); palmitic acid (45% on average) was found to be the main FA, followed by stearic (20% on average), linoleic (17% on average), and alpha-linolenic (13% on average) acids, while the proportion of SFA was found to be the highest among others [47].

### 3.2. Parameters of Non-Treated and Fermented Bovine Colostrum

**Figure 6:**
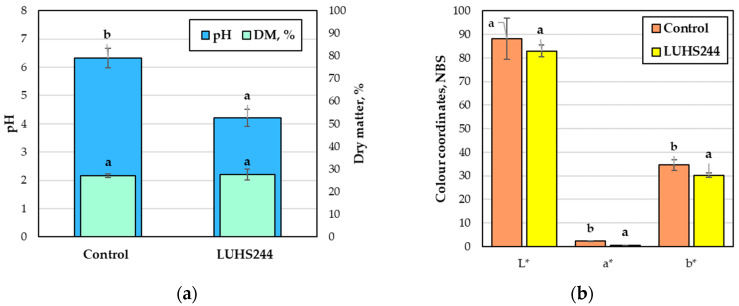
Bovine colostrum (**a**) pH and dry matter (DM), (**b**) colour coordinates (L*—lightness; a*—redness or −a*—greenness; b*—yellowness or −b*—blueness). Control—non-treated bovine colostrum; LUHS244—bovine colostrum fermented with *Lacticaseibacillus paracasei* LUHS244; NBS—National Bureau of Standards units; DM—dry matter. Data are represented as means (n = 3) ± standard error. a–b mean values within a line with different letters are significantly different (*p* ≤ 0.05).

The pH value, dry matter, and colour coordinates of the bovine colostrum are provided in Figure 6. The dry matter of the non-treated and fermented colostrum was similar and reached 27.3% on average (Figure 6a). Fermentation with *L. paracasei* decreased the pH of the colostrum by 33.4%. The lightness (L*) values of the non-treated and fermented colostrum were similar (on average, 85.6 NBS) (Figure 6b). However, the values of the b* and a* coordinates were lower by 12.7% and 5.4 times, respectively, compared to the non-treated samples.

The pH of bovine colostrum ranges from 6.0 to 6.61, and it is suggested that the higher levels of dihydrogen phosphate, citrate, protein, and carbon dioxide contribute to the low pH value [48]. In this study, the obtained pH and dry matter results of the non-treated bovine colostrum are similar with those reported in other studies [49,50]. During fermentation, a decrease in pH is brought on by the bacterial production of organic acids, particularly lactic acid [23]. The yellowness in the colour of bovine colostrum is elicited by the higher levels of carotenoids (lutein, all-*trans* β-carotene, and *cis*-13 β-carotene), while the redness may occur due to the presence of red blood cells [48]. It was also reported that over time, the value of the L* coordinate increases and the b* and a* coordinates decrease in bovine colostrum. Bound pigments may be released because of the pH drop and increased enzyme activity during the fermentation process, which may account for the changes in the colour coordinates of fermented colostrum [23].

**Figure 7:**
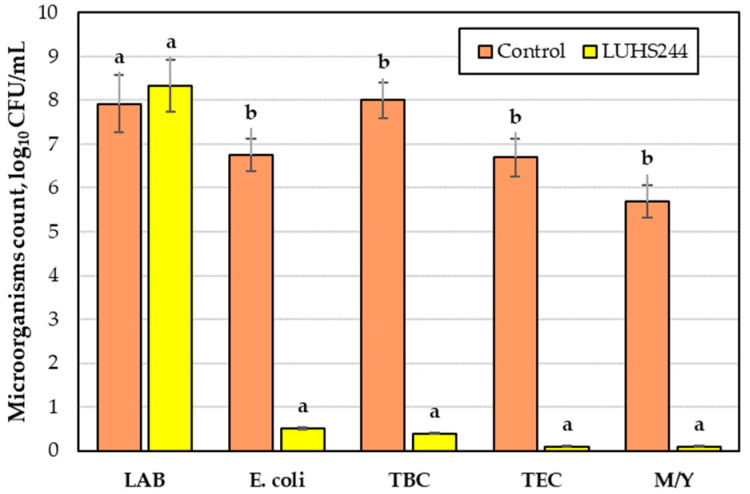
Bovine colostrum microbiological parameters. LAB—lactic acid bacteria; *E. coli*—*Escherichia coli*; TBC—total bacteria count; TEC—total enterobacteria count, M/Y—mould and yeast, Control—non-treated bovine colostrum, LUHS244—bovine colostrum fermented with *Lacticaseibacillus paracasei* LUHS244. Data are represented as means (n = 3) ± standard error; a–b mean values within a line with different letters are significantly different (*p* ≤ 0.05).

The microbiological parameters of bovine colostrum, both non-treated and fermented with *Lacticaseibacillus paracasei* LUHS244, are provided in Figure 7. The total LAB counts between the non-treated and fermented colostrum were similar. However, after fermentation, the *Escherichia coli*, total bacteria, total enterobacteria, and total mould/yeast counts were significantly lower, on average, by 13.2-, 20-, 67-, and 57-fold, respectively.

Microorganisms in bovine colostrum may appear from the mammary gland or during the collection, manipulation, and storage of colostrum [51]. Our findings for the non-treated colostrum slightly contrast with those of Santos et al., who found that the LAB, enterobacteria, and yeast counts of bovine colostrum from three different commercial dairy farms in Brazil accounted for 4.5–5.1, 1.4–3.5, and 1.3–1.7 log_10_ CFU/mL, respectively [52]. However, these differences between studies may arise because of variances in geography, climate, technology, management practices, and degree of hygiene [51]. The presence of LAB in colostrum is common, and the growth rate of these bacteria rapidly increases due to unfit storage conditions and temperature [52]. LAB break down lactose and reduce pH, while their probiotic properties are also beneficial to the animal immune system. The presence of *Enterobacteria* is usually related to the lack of hygiene before, during, and after colostrum milking [52]. Bovine colostrum may contain a diverse range of *E. coli* strains, but just a few strains are harmful, and others do not elicit infections [53]. The process of fermentation with LAB allows for the biological preservation of bovine colostrum [53]. The synthesis of numerous antibacterial substances by *Lactobacillus* strains is well-known, and their antimicrobial properties are influenced by their environment of growth [54]. In our study, the reduced contamination of fermented bovine colostrum could be related to the ability of LAB to produce inhibitory compounds (acetic and lactic acids, hydrogen peroxide, and others) and to synthesize antimicrobial peptides (bacteriocins) [55]. The high antimicrobial activity of *Lacticaseibacillus paracasei LUHS244* was already reported in our previous research [31].

### 3.3. Overall Acceptability, Colour, and Texture Parameters of the Separate Nutraceutical Layers and Overall Acceptability of the Whole Nutraceutical

Images of the produced nutraceutical layers and the final product are provided in Table 2.

The colour coordinates and texture hardness of the separate nutraceutical layers are provided in Table 3. The values of the layer colour coordinates depended on the colour of the main ingredients (fermented Spirulina, fermented bovine colostrum, Jerusalem artichoke, and apple cider vinegar) used for each layer. However, the colour coordinates of layers with same main ingredients were not influenced by the type of sweetener (sugar or xylitol), except for the L* coordinate of the V and VI layers (with apple cider vinegar), and the b* coordinate of the I and II layers (with fermented Spirulina). The lowest values of the L*, a*, and b* coordinates were found for layers containing fermented Spirulina (formulations I and II). The highest lightness and yellowness were determined for layers with apple cider vinegar (formulations V and VI). Coordinate a* was the highest for layers with bovine colostrum and Jerusalem artichoke (formulations III and IV).

The analysis of texture hardness showed that layers with apple cider vinegar were the softest, while layers with bovine colostrum and Jerusalem artichoke (formulation III) had the highest hardness.

According to the evaluation of the overall acceptability of the separate layers, layers with fermented Spirulina received the highest scores, while the lowest acceptability was found for layers with apple cider vinegar (Table 3). The final nutraceuticals were evaluated the same as layers with fermented Spirulina and received the highest score. The acceptability of layers with the same main ingredients were not influenced by the type of sweetener (sugar or xylitol).

The lower hardness of layers with apple cider vinegar could be explained by the fact that the acids in vinegar induce gelatine hydrolysis and reduce its ability to gel in water [56]. Moreover, the incorporation of milk proteins affects the hardness of gelatine gels by stabilizing them [7]. That is why layers with bovine colostrum had the highest hardness in our study. However, it was reported that functional foods such as yogurts, curd, cheese, ice creams, kefir, milk-based beverages, and candies with the inclusion of the bovine colostrum showed sensory acceptance and nutritional benefits [16,57,58]. Spirulina is mainly incorporated into cereal-based products (biscuits, pasta, and bread), snacks, dairy products, and as a potential meat substitute [59]. However, the high level of Spirulina inclusion in foods can cause undesirable changes in physicochemical quality parameters and texture and a lower acceptance [59]. Fermentation with LAB influences the taste and odour of foods and therefore improves the sensory properties of fermented food [60]. During fermentation, lipolysis and proteolysis are induced and small peptides, free fatty acids, and amino acids are generated, which act as flavour precursors [61]. As flavour is a key element in consumer acceptance, this could probably explain the high acceptability scores of the nutraceuticals produced in our study. To the best of our knowledge, compositions of nutraceuticals such as those included in our study have not been tested by other researchers. However, other bioactive ingredients were used for functional gummy preparation. Paternina et al. prepared gummies enriched with Spirulina biomass and freeze-dried acai pulp and observed that the acceptability index was satisfactory for these candies [8]. Kumkong et al. formulated gummies with a whey protein concentrate and freeze-dried aril and pulp from Gac fruit, which is natural colourant and is beneficial to health [6]. Niam et al. proposed a chewable gummy formulation of Bastard Cedar leaves (*Guazuma Ulmifolia*), Senna leaves (*Cassia Angustifolia*) and lime extracts for a low-calorie diet [9]. Rani et al. produced chewable gummy tablets with *Moringa oleifera* leaf powder, which possess high antioxidant activity, using two types of gelling agents [10].

## 4. Conclusions

In this study, the formulation of multifunctional nutraceuticals based on Spirulina powder, bovine colostrum, Jerusalem artichoke powder, and apple cider vinegar was proposed. The nutraceuticals comprised three layers: (I) a layer with fermented Spirulina; (II) a layer with fermented bovine colostrum and Jerusalem artichoke powder; (III) a layer with apple cider vinegar. The fermentation of Spirulina and bovine colostrum with the strains *Pediococcus acidilactici* No. 29 and *Lacticaseibacillus paracasei* LUHS244, respectively, which was carried out before nutraceutical preparation, significantly influenced most of the tested parameters of these ingredients. In comparison to the untreated Spirulina, the fermented samples had higher concentration of gamma-aminobutyric and L-glutamic acids (by 5.2 and 31.4%, respectively). The fatty acid profile of the fermented Spirulina showed increased alpha-linolenic, gamma-linolenic, and omega-3 fatty acid contents. The fermentation of bovine colostrum significantly improved its microbiological safety. The colour coordinates of the separate nutraceutical layers depended on the colour of the main ingredients. The hardest layers were those made with Jerusalem artichoke and bovine colostrum, whereas the softest layers were those made with apple cider vinegar. The results of the current study indicate a significant potential of the suggested combination for a nutraceutical with enhanced functionality and a high rating for acceptability.

## Figures and Tables

**Table 1:** Formulas for the layers of nutraceuticals.

Ingredients	Formulas for Layers					
	I	II	III	IV	V	VI
Sugar, g	17	-	17	-	17	-
Xylitol, g	-	17	-	17	-	17
Gelatine, g	8.5	8.5	8.5	8.5	8.5	8.5
Citric acid, g	0.7	0.7	0.7	0.7	-	-
Fermented Spirulina powder, g	5	5	-	-	-	-
Water, mL	20	20	20	20	20	20
*C. paradise* essential oil, µL	2	2	-	-	-	-
Fermented bovine colostrum, g	-	-	7.0	7.0	-	-
Jerusalem artichoke powder, g	-	-	5.0	5.0	-	-
Apple cider vinegar, mL	-	-	-	-	15	15

**Table 2:** Images of the separate nutraceutical layers and whole product.

Nutraceutical Formulations	Images
Formulation I (sugar + gelatine + citric acid + fermented Spirulina + water + *C. paradise* essential oil)	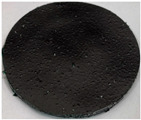
Formulation II (xylitol + gelatine + citric acid + fermented Spirulina + water + *C. paradise* essential oil)	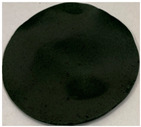
Formulation III (sugar + gelatine + citric acid + fermented bovine colostrum + Jerusalem artichoke powder + water)	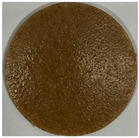
Formulation IV (xylitol + gelatine + citric acid + fermented bovine colostrum + Jerusalem artichoke powder + water)	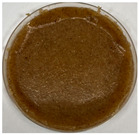
Formulation V (sugar + gelatine + apple cider vinegar + water)	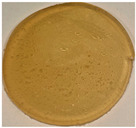
Formulation VI (xylitol + gelatine + apple cider vinegar + water)	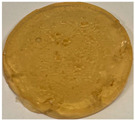
Whole (a) and cut (b) nutraceutical (a) (Layers I + III + V and Layers II + IV + VI)	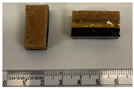 (a) 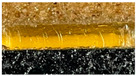 (b)

**Table 3:** Colour coordinates and texture hardnesses of the separate nutraceutical layers and overall acceptability of the separate layers and final products.

NutraceuticalFormulations	Colour Coordinates, NBS			Texture Hardness, mJ	Overall Acceptability
	L*	a*	b*		
I	26.5 ± 0.3 a	−1.03 ± 0.06 a	0.98 ± 0.05 a	1.20 ± 0.18 bc	10.0 ± 0.3 b
II	28.7 ± 0.7 a	−0.90 ± 0.05 a	2.25 ± 0.18 b	0.90 ± 0.11 b	10.0 ± 0.5 b
III	50.6 ± 0.6 b	4.49 ± 0.46 c	14.10 ± 0.31 c	1.70 ± 0.12 d	8.5 ± 0.7 a
IV	47.0 ± 0.8 b	5.35 ± 0.79 c	13.01 ± 0.58 c	1.30 ± 0.15 c	8.5 ± 0.8 a
V	71.4 ± 0.9 d	0.65 ± 0.15 b	20.50 ± 0.42 d	0.30 ± 0.19 a	8.0 ± 0.4 a
VI	63.9 ± 1.2 c	1.11 ± 0.25 b	20.20 ± 0.27 d	0.50 ± 0.16 a	8.0 ± 0.6 a
I + III + V	-	-	-	-	10.0 ± 0.5 b
II + IV + VI	-	-	-	-	10.0 ± 0.7 b

L*—lightness; a*—redness or—a*—greenness; b*—yellowness or—b*—blueness; NBS—National Bureau of Standards units; I—Formulation I (sugar + gelatine + citric acid + fermented Spirulina + water+ *C. paradise* essential oil); II—Formulation II (xylitol + gelatine + citric acid + fermented Spirulina + water+ *C. paradise* essential oil); III—Formulation III (sugar + gelatine + citric acid + fermented bovine colostrum + Jerusalem artichoke powder + water); IV—Formulation IV (xylitol + gelatine + citric acid + fermented bovine colostrum + Jerusalem artichoke powder + water); V—Formulation V (sugar + gelatine + apple cider vinegar + water); VI—Formulation VI (xylitol + gelatine + apple cider vinegar + water); I + III + V and II + IV + VI—the final products. a–d mean values denoted with different letters indicate significantly different values between the samples (*p* ≤ 0.05).

## Data Availability

The data are available from the corresponding author upon reasonable request.

## References

[1] Dima C., Assadpour E., Dima S., Jafari S.M. (2020). Bioavailability of Nutraceuticals: Role of the Food Matrix, Processing Conditions, the Gastrointestinal Tract, and Nanodelivery Systems. Compr. Rev. Food Sci. Food Saf..

[2] Parmar K., Patel J. (2019). Natural Flavors in Various Nutraceutical Product Applications. Flavor Development for Functional Foods and Nutraceuticals.

[3] Santini A. (2022). Nutraceuticals and Functional Foods: Is It Possible and Sustainable for Bridging Health and Food?. Foods.

[4] Daliu P., Santini A., Novellino E. (2019). From Pharmaceuticals to Nutraceuticals: Bridging Disease Prevention and Management. Expert Rev. Clin. Pharmacol..

[5] Grand View Research (2017). Nutraceuticals Market Analysis by Product (Dietary Supplements, Function-al Food, Functional Beverage), by Region (North America, Asia Pacific, Europe, CSA, MEA), and Segment Forecasts, 2018–2025.

[6] Kumkong N., Banjongsinsiri P., Laohakunjit N., Vatanyoopaisarn S., Thumthanaruk B. (2020). Influence of Natural Colour Blends of Freeze-Dried Gac Aril and Pulp on the Quality of Whey Protein-Mixed Gelatin-Based Chewables. Heliyon.

[7] Yan B., Davachi S.M., Ravanfar R., Dadmohammadi Y., Deisenroth T.W., Van Pho T., Odorisio P.A., Darji R.H., Abbaspourrad A. (2020). Improvement of vitamin C stability in vitamin gummies by encapsulation in casein gel. Food Hydrocoll..

[8] Paternina L.P.R., Moraes L., Santos T.D., de Morais M.G., Costa J.A.V. (2022). Spirulina and Açai as Innovative Ingredients in the Development of Gummy Candies. J. Food Process. Preserv..

[9] Niam M.L.Q., Amin R.S., Utami N., Wahyuni A.S. (2022). Formulation of Dietary Supplement Chewable Gummy with Bastard Cedar Leaves (*Guazuma Ulmifolia*), Senna Leaves (*Cassia Angustifolia*) and Lime Extracts Using a Simplex Lattice Design. Proceedings of the International Conference on Sustainable Innovation on Health Sciences and Nursing ICOSI-HSN.

[10] Rani K.C., Jayani N.I.E., Feneke F., Melanda S. (2021). Preparation and evaluation of gelatin and pectin-based moringa oleifera chewable-gummy tablets. Proceedings of the IOP Conference Series: Earth and Environmental Science.

[11] de la Jara A., Ruano-Rodriguez C., Polifrone M., Assunçao P., Brito-Casillas Y., Wägner A.M., Serra-Majem L. (2018). Impact of Dietary Arthrospira (Spirulina) Biomass Consumption on Human Health: Main Health Targets and Systematic Review. J. Appl. Phycol..

[12] Priyanka S., Varsha R., Verma V., Ayenampudi S.B. (2023). Spirulina: A Spotlight on Its Nutraceutical Properties and Food Processing Applications. J. Microbiol. Biotechnol. Food Sci..

[13] Kumaraguruparaswami M., Subramani D., Arunachalam S., Kandasamy S., Sivasubramaniyan S.G., Nallamuthu D. (2022). Spirulina-derived nutraceuticals and their applications in the food industry. Algal Genet. Resour. Cosmeceuticals Nutraceuticals Pharm. Algae.

[14] Playford R.J., Weiser M.J. (2021). Bovine Colostrum: Its Constituents and Uses. Nutrients.

[15] Gomes R.D., Anaya K., Galdino A.B., Oliveira J.P., Gama M.A., Medeiros C.A., Gavioli E.C., Porto A.L.F., Rangel A.H. (2021). Bovine Colostrum: A Source of Bioactive Compounds for Prevention and Treatment of Gastrointestinal Disorders. NFS J..

[16] Mehra R., Garhwal R., Sangwan K., Guiné R.P.F., Lemos E.T., Buttar H.S., Visen P.K.S., Kumar N., Bhardwaj A., Kumar H. (2022). Insights into the Research Trends on Bovine Colostrum: Beneficial Health Perspectives with Special Reference to Manufacturing of Functional Foods and Feed Supplements. Nutrients.

[17] Méndez-Yáñez A., Ramos P., Morales-Quintana L. (2022). Human Health Benefits through Daily Consumption of Jerusalem Artichoke (*Helianthus tuberosus* L.) Tubers. Horticulturae.

[18] Rubel I.A., Iraporda C., Novosad R., Cabrera F.A., Genovese D.B., Manrique G.D. (2018). Inulin Rich Carbohydrates Extraction from Jerusalem Artichoke (*Helianthus tuberosus* L.) Tubers and Application of Different Drying Methods. Food Res. Int. Ott. Ont.

[19] Rahim M.A., Saeed F., Khalid W., Hussain M., Anjum F.M. (2021). Functional and Nutraceutical Properties of Fructo-Oligosaccharides Derivatives: A Review. Int. J. Food Prop..

[20] Tripathi S. (2020). Apple Cider Vinegar (ACV) and Their Pharmacological Approach towards Alzheimer’s Disease (AD): A Review. Indian J. Pharm. Educ. Res..

[21] Shirazi A.O., Jahandideh H., Yarahmadi A., Milanifard M., Delarestaghi M.M., Maleki M. (2020). The Effect of Apple Cider Vinegar in the Treatment of Chronic Rhinosinusitis. Med. Sci..

[22] Taroncher M., Vila-Donat P., Tolosa J., Ruiz M.J., Rodríguez-Carrasco Y. (2021). Biological Activity and Toxicity of Plant Nutraceuticals: An Overview. Curr. Opin. Food Sci..

[23] de Marco Castro E., Shannon E., Abu-Ghannam N. (2019). Effect of Fermentation on Enhancing the Nutraceutical Properties of Arthrospira Platensis (Spirulina). Fermentation.

[24] Wang Y., Wu J., Lv M., Shao Z., Hungwe M., Wang J., Bai X., Xie J., Wang Y., Geng W. (2021). Metabolism Characteristics of Lactic Acid Bacteria and the Expanding Applications in Food Industry. Front. Bioeng. Biotechnol..

[25] Cui Y., Miao K., Niyaphorn S., Qu X. (2020). Production of Gamma-Aminobutyric Acid from Lactic Acid Bacteria: A Systematic Review. Int. J. Mol. Sci..

[26] Jia M., Zhu Y., Wang L., Sun T., Pan H., Li H. (2022). PH Auto-Sustain-Based Fermentation Supports Efficient Gamma-Aminobutyric Acid Production by Lactobacillus Brevis CD0817. Fermentation.

[27] Sahab N.R.M., Subroto E., Balia R.L., Utama G.L. (2020). γ-Aminobutyric Acid Found in Fermented Foods and Beverages: Current Trends. Heliyon.

[28] Tolpeznikaite E., Bartkevics V., Skrastina A., Pavlenko R., Ruzauskas M., Starkute V., Zokaityte E., Klupsaite D., Ruibys R., Rocha J.M. (2023). Submerged and Solid-State Fermentation of Spirulina with Lactic Acid Bacteria Strains: Antimicrobial Properties and the Formation of Bioactive Compounds of Protein Origin. Biology.

[29] Bartkiene E., Lele V., Sakiene V., Zavistanaviciute P., Ruzauskas M., Stankevicius A., Grigas J., Pautienius A., Bernatoniene J., Jakstas V. (2020). Fermented, Ultrasonicated, and Dehydrated Bovine Colostrum: Changes in Antimicrobial Properties and Immunoglobulin Content. J. Dairy Sci..

[30] Bartkiene E., Bartkevics V., Ikkere L.E., Pugajeva I., Zavistanaviciute P., Lele V., Ruzauskas M., Bernatoniene J., Jakstas V., Klupsaite D. (2018). The Effects of Ultrasonication, Fermentation with *Lactobacillus* Sp., and Dehydration on the Chemical Composition and Microbial Contamination of Bovine Colostrum. J. Dairy Sci..

[31] Bartkiene E., Lele V., Ruzauskas M., Domig K.J., Starkute V., Zavistanaviciute P., Bartkevics V., Pugajeva I., Klupsaite D., Juodeikiene G. (2020). Lactic Acid Bacteria Isolation from Spontaneous Sourdough and Their Characterization Including Antimicrobial and Antifungal Properties Evaluation. Microorganisms.

[32] Tolpeznikaite E., Bartkevics V., Skrastina A., Pavlenko R., Mockus E., Zokaityte E., Starkute V., Klupsaite D., Ruibys R., Rocha J.M. (2023). Changes in Spirulina’s Physical and Chemical Properties during Submerged and Solid-State Lacto-Fermentation. Toxins.

[33] (2017). Sensory Analysis—Methodology—General Guidance 2017.

[34] Park W.S., Kim H.-J., Li M., Lim D.H., Kim J., Kwak S.-S., Kang C.-M., Ferruzzi M.G., Ahn M.-J. (2018). Two Classes of Pigments, Carotenoids and c-Phycocyanin, in Spirulina Powder and Their Antioxidant Activities. Molecules.

[35] Kurt H., Isleten Hosoglu M., Guneser O., Karagul-Yuceer Y. (2023). Influence of Different Bacteria Species in Chemical Composition and Sensory Properties of Fermented Spirulina. Food Chem..

[36] Bao J., Zhang X., Zheng J.-H., Ren D.-F., Lu J. (2018). Mixed Fermentation of Spirulina Platensis with Lactobacillus Plantarum and Bacillus Subtilis by Random-Centroid Optimization. Food Chem..

[37] Cebi N., Dogan C.E., Olgun E.O., Sagdic O. (2018). A Survey of Free Glutamic Acid in Foods Using a Robust LC–MS/MS Method. Food Chem..

[38] Woraharn S., Lailerd N., Sivamaruthi B.S., Wangcharoen W., Sirisattha S., Peerajan S., Chaiyasut C. (2016). Evaluation of Factors That Influence the L-Glutamic and γ-Aminobutyric Acid Production during Hericium Erinaceus Fermentation by Lactic Acid Bacteria. CyTA-J. Food.

[39] Alizadeh Behbahani B., Jooyandeh H., Falah F., Vasiee A. (2020). Gamma-aminobutyric Acid Production by Lactobacillus Brevis A3: Optimization of Production, Antioxidant Potential, Cell Toxicity, and Antimicrobial Activity. Food Sci. Nutr..

[40] Anggraini L., Marlida Y., Wizna W., Jamsari J., Mirzah M. (2019). Optimization of Nutrient Medium for Pediococcus Acidilactici DS15 to Produce GABA. J. Worlds Poult. Res..

[41] Thuy D.T.B., Nguyen A.T., Khoo K.S., Chew K.W., Cnockaert M., Vandamme P., Ho Y.-C., Huy N.D., Cocoletzi H.H., Show P.L. (2021). Optimization of Culture Conditions for Gamma-Aminobutyric Acid Production by Newly Identified Pediococcus Pentosaceus MN12 Isolated from ‘Mam Nem’, a Fermented Fish Sauce. Bioengineered.

[42] Matos J., Cardoso C.L., Falé P., Afonso C.M., Bandarra N.M. (2020). Investigation of Nutraceutical Potential of the Microalgae Chlorella Vulgaris and Arthrospira Platensis. Int. J. Food Sci. Technol..

[43] Grosshagauer S., Kraemer K., Somoza V. (2020). The True Value of Spirulina. J. Agric. Food Chem..

[44] AlFadhly N.K., Alhelfi N., Altemimi A.B., Verma D.K., Cacciola F., Narayanankutty A. (2022). Trends and Technological Advancements in the Possible Food Applications of Spirulina and Their Health Benefits: A Review. Molecules.

[45] Khubber S., Marti-Quijal F.J., Tomasevic I., Remize F., Barba F.J. (2022). Lactic Acid Fermentation as a Useful Strategy to Recover Antimicrobial and Antioxidant Compounds from Food and By-Products. Curr. Opin. Food Sci..

[46] Filannino P., Di Cagno R., Gobbetti M. (2018). Metabolic and Functional Paths of Lactic Acid Bacteria in Plant Foods: Get out of the Labyrinth. Curr. Opin. Biotechnol..

[47] Dewi E.N., Amalia U. (2018). Nutritional Comparison of Spirulina Sp Powder by Solid-State Fermentation Using Aspergillus Sp (FNCL 6088) and Lactobacillus Plantarum (FNCL 0127). Proceedings of the IOP Conference Series: Earth and Environmental Science.

[48] McGrath B.A., Fox P.F., McSweeney P.L., Kelly A.L. (2016). Composition and Properties of Bovine Colostrum: A Review. Dairy Sci. Technol..

[49] Dande N.D., Nande P.J. (2020). Nutritional Composition of Bovine Colostrum: Palatability Evaluation of Food Products Prepared Using Bovine Colostrum. Int. J. Nutr. Pharmacol. Neurol. Dis..

[50] Puppel K., Gołębiewski M., Grodkowski G., Slósarz J., Kunowska-Slósarz M., Solarczyk P., Łukasiewicz M., Balcerak M., Przysucha T. (2019). Composition and Factors Affecting Quality of Bovine Colostrum: A Review. Animals.

[51] Šlosárková S., Pechová A., Staněk S., Fleischer P., Zouharová M., Nejedlá E. (2021). Microbial Contamination of Harvested Colostrum on Czech Dairy Farms. J. Dairy Sci..

[52] Dos Santos G., Da Silva J.T., da Rocha Santos F.H., Bittar C.M.M. (2017). Nutritional and Microbiological Quality of Bovine Colostrum Samples in Brazil. Rev. Bras. Zootec..

[53] Fasse S., Alarinta J., Frahm B., Wirtanen G. (2021). Bovine Colostrum for Human Consumption—Improving Microbial Quality and Maintaining Bioactive Characteristics through Processing. Dairy.

[54] Mörschbächer A.P., Granada C.E. (2022). Mapping the Worldwide Knowledge of Antimicrobial Substances Produced by Lactobacillus Spp.: A Bibliometric Analysis. Biochem. Eng. J..

[55] Saalfeld M.H., Pereira D.I.B., Valente J.D.S.S., Borchardt J.L., Weissheimer C.F., Gularte M.A., Leite F.P.L. (2016). Effect of Anaerobic Bovine Colostrum Fermentation on Bacteria Growth Inhibition. Ciênc. Rural.

[56] Wang R., Hartel R.W. (2022). Citric Acid and Heating on Gelatin Hydrolysis and Gelation in Confectionery Gels. Food Hydrocoll..

[57] Bagwe S., Tharappel L.J., Kaur G., Buttar H.S. (2015). Bovine Colostrum: An Emerging Nutraceutical. J. Complement. Integr. Med..

[58] Kaplan M., Arslan A., Duman H., Karyelioğlu M., Baydemir B., Günar B.B., Alkan M., Bayraktar A., Tosun H.İ., Ertürk M. (2022). Production of Bovine Colostrum for Human Consumption to Improve Health. Front. Pharmacol..

[59] Lafarga T., Fernández-Sevilla J.M., González-López C., Acién-Fernández F.G. (2020). Spirulina for the Food and Functional Food Industries. Food Res. Int..

[60] Laaksonen O., Kahala M., Marsol-Vall A., Blasco L., Järvenpää E., Rosenvald S., Virtanen M., Tarvainen M., Yang B. (2021). Impact of Lactic Acid Fermentation on Sensory and Chemical Quality of Dairy Analogues Prepared from Lupine (*Lupinus angustifolius* L.) Seeds. Food Chem..

[61] Hu Y., Zhang L., Wen R., Chen Q., Kong B. (2022). Role of Lactic Acid Bacteria in Flavor Development in Traditional Chinese Fermented Foods: A Review. Crit. Rev. Food Sci. Nutr..

